# Examining mechanisms by which acute psychosocial stress disrupts the proestrous LH surge and ovulation in female mice

**DOI:** 10.1210/endocr/bqag070

**Published:** 2026-06-15

**Authors:** Elizabeth R Wagenmaker, Amanda G Gibson, Megan S Johnson, Abira A Choudhry, Nicholas M Provenzano, Bo Dong, Suzanne M Moenter

**Affiliations:** Departments of Molecular & Integrative Physiology, University of Michigan, Ann Arbor, MI 48109, USA; Departments of Molecular & Integrative Physiology, University of Michigan, Ann Arbor, MI 48109, USA; Neuroscience Graduate Program, University of Michigan, Ann Arbor, MI 48109, USA; Departments of Molecular & Integrative Physiology, University of Michigan, Ann Arbor, MI 48109, USA; Departments of Molecular & Integrative Physiology, University of Michigan, Ann Arbor, MI 48109, USA; Departments of Molecular & Integrative Physiology, University of Michigan, Ann Arbor, MI 48109, USA; Departments of Molecular & Integrative Physiology, University of Michigan, Ann Arbor, MI 48109, USA; Departments of Molecular & Integrative Physiology, University of Michigan, Ann Arbor, MI 48109, USA; Internal Medicine, University of Michigan, Ann Arbor, MI 48109, USA; Obstetrics & Gynecology, University of Michigan, Ann Arbor, MI 48109, USA; The Reproductive Sciences Program, University of Michigan, Ann Arbor, MI 48109, USA

**Keywords:** luteinizing hormone, ovulation, stress, adrenal gland

## Abstract

Stress most often inhibits reproduction. Exposure to an acute layered psychosocial stress paradigm (ALPS) on proestrus disrupts the LH surge in 60% to 70% of adult female mice without interfering with the progression of the estrous cycle. The present study aims to expand on these observations by investigating effects of ALPS exposure on ovulation and potential mechanisms involved in surge disruption. The most common response to ALPS exposure was disruption of the LH surge and blockade of ovulation; however, in some mice in which no LH surge was detected, ovulation did occur. Factors from the adrenal cortex and medulla, corticosterone and catecholamines (norepinephrine and epinephrine), respectively, are increased during stress; we thus tested whether these factors mediate the effects of stress on the LH surge and ovulation by applying ALPS in adrenalectomized (ADX) animals. ADX did not reverse the disruptions of the LH surge/ovulation after ALPS. Further studies aimed to investigate potential roles of individual adrenal factors in this response. We tested whether administering corticosterone, in the absence of stress, was sufficient to suppress the LH surge and ovulation. It was not. Finally, we tested whether blockade of β-adrenergic signaling during ALPS would rescue the LH surge and/or ovulation. Propranolol, a nonselective β-adrenergic receptor blocker, given 1 hour before ALPS failed to reverse the disruptive effects of stress. Together, these data confirm the effectiveness of ALPS in disrupting both the LH surge and ovulation. They further indicate that the adrenal gland is dispensable for these effects.

The female reproductive cycle is controlled at the level of the hypothalamus, with highly regulated GnRH release leading to gonadotropin synthesis and secretion from the anterior pituitary and, ultimately, production of sex steroids in the ovary (1). These sex steroids provide feedback control to regulate GnRH and gonadotropin release. During most of the female reproductive cycle, steroid negative feedback modulates pulsatile release of these hormones. However, during the late follicular phase (proestrus in rodents), estradiol exerts positive feedback, leading to sustained surges in GnRH and LH release that ultimately result in ovulation (2-6). Disruptions at any level of this hormonal axis could alter or even prevent ovulation, leading to reduced fertility.

One such disrupting factor is stress. Psychosocial stressors, such as social isolation and restraint, suppress GnRH and/or LH secretion in many species, including sheep, primates, and rodents (7-9), but evidence of effects on ovulation is lacking. Exposure of proestrous female mice to an acute layered psychosocial stress (ALPS) paradigm consisting of sequential social isolation, transportation, restraint, and predator stress beginning 6.5 hours after lights on disrupts the proestrous LH surge in most animals, at least in part, by interfering with estradiol positive feedback (10). Whether these animals ovulate and the physiological mechanisms underlying the disruption remain unknown.

Acute stress increases secretion of adrenal factors, such as glucocorticoids from the cortex and the catecholamines epinephrine and norepinephrine (NE) from the medulla. Glucocorticoids, in the absence of stress, suppress pulsatile LH secretion in a variety of species (11). Glucocorticoid suppression of LH pulses in many of these studies required long-term glucocorticoid administration (typically >24 hours) and the presence of estradiol (12, 13). Studies investigating acute effects of stress-like levels of glucocorticoids on the LH surge, which is induced by sustained elevated estradiol levels, and ovulation are lacking. Catecholamines can also regulate the hypothalamic-pituitary-ovarian axis. NE in particular plays a critical stimulatory role in LH surge initiation in rodents (14), is highly regulated by estradiol (15), and could be a potential mediator of stress-induced disruptions of the LH surge mechanism.

We conducted several experiments aimed to test the hypothesis that a single exposure to psychosocial stress on proestrus is sufficient to disrupt not only the preovulatory LH surge but also ovulation in adult female mice. We also investigated possible mechanisms by which stress is conveyed to the reproductive neuroendocrine axis, including the potential roles of the stress-related adrenal factors, corticosterone and catecholamines.

## Materials and methods

### Animals

Adult (aged 61-163 days) female CBA/B6F1 mice expressing green fluorescent protein under control of the GnRH promoter [Tg(Gnrh1-EGFP)51Sumo MGI:6158457] (16) were used for all experiments. All mice were provided with water and Teklad 2916 chow (Envigo, Madison, WI, USA) ad libitum and were housed on a 14L:10D light cycle with lights on at 0300 EST. Estrous cycle stage was determined via vaginal cytology and was monitored for at least 7 days before studies began. Some mice were bilaterally adrenalectomized (ADX) or underwent sham surgery. Surgery was done under isoflurane general anesthesia with carprofen analgesic [5 mg/kg, subcutaneously (s.c.); 30 minutes before and 24 hours postsurgery (Zoetis Inc, Kalamazoo, MI)]. ADX mice were provided with 0.9% NaCl drinking water. Studies were performed at least 10 days after surgery to allow for complete recovery; estrous cycles were monitored starting 7 days postsurgery and were not disrupted by surgery. The Institutional Animal Care and Use Committee of the University of Michigan approved all procedures.

### Acute layered psychosocial stress paradigm

To test whether acute psychosocial stress affects ovulation, animals were exposed to an acute layered stress paradigm on proestrus (starting 6.5 hours after lights on) (10). Mice were placed in a new cage and moved to a new room, followed 1 hour later by placement in a restraint device; mice were able to turn around within the device. Two hours after restraint began, the predator odor 2,3,5-trimethyl-3-thiazoline (Contech Enterprises; Victoria, BC, Canada), a component of red fox (*Vulpes vulpes*) urine, was introduced to the cage for another 2 hours for a total of 5 hours of exposure to psychosocial stress. Nonstressed control mice remained in their home cage for the duration of the experiment. In the following discussion, these groups are referred to as exposed to control or ALPS conditions.

### Blood sampling

LH and corticosterone were measured at multiple time points from tail blood samples. To minimize stress during blood sampling, all samples were taken in a quiet, calm environment and mice were habituated to the handling procedures for ≥2 weeks before studies. Mice were gently removed from their cage and placed on a work surface. While lightly holding the tail, the tip of the tail (<2 mm) was nicked with a sterile scalpel blade. Animals were allowed as much freedom of movement as possible while collecting tail blood via capillary tube for corticosterone (25-40 µL/sample) or Pipetman (6 µL/sample) for LH measurement. Blood for corticosterone measurement was centrifuged and serum stored at −20 °C until assay. Whole blood for LH determination was immediately mixed with 54 µL cold assay buffer (0.2% BSA [Jackson ImmunoResearch] and 0.05% TWEEN-20 [Sigma] in 7.4 pH PBS [GIBCO]) and kept on ice for up to 3 hours before being stored at −20 °C until assay. Sampling for LH measurement after lights out was done under dim (<5 lux) red light.

### Oral drug administration

Injection and the associated restraint can induce stress. To alleviate this potential complicating factor, particularly in studies in which drugs are given at multiple time points (experiment 5), mice were administered drugs in Nutella when possible as previously described (17). At the start of studies, animals were pair-housed, and the bottom of a 50 mm Petri dish was placed in the cage. For at least 1 week before the experiment, animals were individually trained to eat ∼60 mg Nutella placed on the dish. One animal was removed from the home cage and placed in a holding cage while the remaining animal was given the opportunity to eat the Nutella. Once the Nutella was ingested, the procedure was repeated for the other mouse. Mice typically ate the Nutella within 30 seconds after 2 or 3 days of training. As this method of administration requires 1 mouse to be moved into a new cage, which elevates serum corticosterone, only 1 animal per cage was used in an experiment on any given day.

### Detection of proestrus and ovulation

LH surges in nocturnal rodents occur on the afternoon of proestrus. Mice with a morning vaginal smear containing primarily nucleated cells were selected for exposure to control or ALPS conditions that day. In past work, uterine mass was used to confirm cycle stage; in this strain, a uterine mass of >125 mg strongly correlates with an LH surge and is thus indicative of proestrus. In the present studies, however, the goal was to examine oviducts for ovulation the next morning (estrus); this precluded removal of the uterus on the afternoon of proestrus. A set of criteria was thus developed for study inclusion. For vehicle-treated nonstressed animals on the day after the experiment: (*a*) vaginal smears were characteristic of estrus (mostly cornified cells), (b) the uterus was pale and fluid-filled, and (*c*) oocytes were associated with cumulus cells and located within a swollen ampulla. All vehicle-treated nonstressed controls met these criteria, and all displayed typically timed LH surges on the afternoon of proestrus and ovulated. For all other treatments, disruption of the LH surge and/or ovulation may occur; thus, only criteria (*a*) and (*b*) could be used to confirm cycle stage at the time of experiments; animals not meeting these 2 criteria were excluded.

To count oocytes, mice were euthanized by carbon dioxide overdose on the morning after exposure to control or ALPS conditions (approximately 16-17 hours after the expected LH surge peak), and the ovary, oviduct, and a small section of the uterus were removed. Tissue was placed in PBS, and oviducts were examined with a dissecting microscope [AXIO Imager M2 (Zeiss; Jena, Germany) or Olympus SZX16 (Evident Scientific; Waltham, MA)]. If a swollen ampulla was observed, a small needle was used to break it open to release the oocytes. If a swollen ampulla was not evident, small needles were used to tease open the length of the oviduct to release any oocytes present. The number of oocytes and their location (ampulla or further down the oviduct) were recorded.

### Experiment 1: Does ALPS disrupt ovulation?

To test the hypothesis that ALPS disrupts ovulation, mice were exposed to control (n = 6) or ALPS conditions (n = 6) on the morning of proestrus. Blood samples were taken immediately before and after stress exposure to measure corticosterone levels; samples were taken at the same time points in nonstressed control mice. To assess the LH surge, blood samples were taken at −2.5 hours (end of the stress exposure) and then hourly from −1 hour to 1 hour relative to lights out. The next morning, estrous cycle stage was determined, mice were euthanized, and the uterus and ovulation were examined as previously described.

### Experiment 2: Does ALPS stimulate LH release resulting in premature ovulation?

Some mice in experiment 1 appeared to ovulate early. To test the hypothesis that stress exposure increases LH on the morning of proestrus, frequent LH measurements were taken in mice exposed to control (n = 8 not frequently sampled for LH; n = 7 bleed control, frequently sampled for LH) or ALPS conditions (n = 6 frequently sampled for LH). Blood samples were taken immediately before and after stress exposure to measure corticosterone levels; samples were taken at these same time points in both control groups. In the frequently sampled bleed control and stress groups, additional samples were taken for LH before stress exposure, then every 15 minutes for 1.5 hours, then every 30 minutes for 1 hour, and then hourly for 2 hours. In all groups, LH was sampled at −2.5, −2, −1, 0, 1, and 2 hours relative to lights off. The next morning, estrous cycle stage was determined, mice were euthanized, and the uterus and ovulation were examined as previously described.

### Experiment 3: Can ALPS exposure induce ovulation in the absence of an LH surge?

To test the hypothesis that ALPS induces ovulation in the absence of an LH surge, proestrous mice were either injected s.c. with sterile saline (vehicle) or 3 mg/kg Antide, a GnRH receptor antagonist (Cayman Chemical), 1 hour before exposure to control or ALPS conditions (n = 6-8/group). Drugs were injected s.c., as Antide is a peptide that could be digested before taking effect; therefore, oral administration via Nutella would not be appropriate. Blood samples for corticosterone measurement were taken immediately before vehicle/Antide administration and before and after stress exposure; samples were taken at the same time points in nonstressed controls. Samples for LH surge detection were taken at hourly intervals from −4 hours to +3 hours relative to lights off. The sampling window for LH measurement was expanded to increase confidence that Antide was effective in blocking the LH surge. The next morning, estrous cycle stage was determined, mice were euthanized, and the uterus and ovulation were examined as previously described.

### Experiment 4: Does adrenalectomy reverse the disruptive effects of ALPS on the LH surge and/or ovulation?

Elevations in adrenal factors are associated with disturbances in the female reproductive system (11, 18, 19). To determine whether these mediate the disruptions in the LH surge and ovulation observed following psychosocial stress exposure, we tested the hypothesis that removal of the adrenal glands blocks these effects.

Female mice were assigned to 1 of 4 treatment groups (n = 12/group): sham-operated nonstress control, sham-operated stress, ADX nonstress control, and ADX stress. Midmorning on the day of proestrus, mice were exposed to control or ALPS conditions. Blood samples were taken immediately before and after stress exposure for corticosterone measurement; samples were taken at the same time points in nonstressed controls. Blood samples for LH measurement were taken at −2.5 hours and hourly from −2 hours to +1 hour or +2 hours relative to lights off; sampling was expanded to +2 hours for the second cohort of mice, as results from the first cohort (n = 6/group) hinted that the LH surge was delayed in the ADX stress group. The next morning, estrous cycle stage was determined, mice were euthanized, and the uterus and ovulation were examined as previously described.

### Experiment 5: Do stress-like levels of corticosterone mimic the effects of ALPS on the LH surge and/or ovulation?

A major component of stress response is a rise in circulating glucocorticoids. We tested the hypothesis that stress-like levels of corticosterone, in the absence of stress, recapitulate the disruptive effects of ALPS exposure on the LH surge and ovulation. Pilot studies were conducted to optimize the protocol for feeding corticosterone (Sigma-Aldrich) in Nutella to produce a serum trajectory that mimics the pattern observed in response to stress exposure. Proestrous mice (n = 12/group) were assigned to vehicle (36% dimethyl sulfoxide) or corticosterone (2 mg/kg in 36% dimethyl sulfoxide) given in approximately 60 mg Nutella at hours 0, 1, and 3; times correspond to changes in the ALPS paradigm. In a pilot study, blood samples taken at hours 0 and 0.5 and hourly from 1 to 5 hours after beginning corticosterone treatment demonstrate that this method resulted in sustained corticosterone levels like those seen during stress exposure. In the main study, blood was sampled for basal corticosterone measurement immediately before first feeding and at the end of the 5-hour treatment period. Blood samples for LH determination were taken from −2.5 hours to +2 hours relative to lights off. The next morning, estrous cycle stage was determined, mice were euthanized, and the uterus and ovulation were examined as previously described.

### Experiment 6: Does blockade of β-adrenergic receptors reverse stress-induced disruption of the LH surge and/or ovulation?

We next tested whether blocking activation of β-adrenergic receptors would ameliorate the disruptive effects of ALPS exposure. Pilot studies were conducted to confirm that the dose of propranolol, a nonselective β-adrenergic receptor antagonist, was sufficient to decrease heart rate in anesthetized mice (%change 10 minutes posttreatment: saline 7.1 ± 6.8% increase, 5 mg/kg propranolol 28.5 ± 6.0% decrease; n = 2/group).

Proestrous mice (n = 12/group) were injected s.c. using light restraint with either vehicle (sterile saline) or 5 mg/kg propranolol (Fresenius Kabi) 1 hour before exposure to control or ALPS conditions. Drugs were not administered orally via Nutella because the mice showed an aversion to the propranolol and would not consistently ingest it. Blood samples were taken immediately before drug treatment and before and after ALPS exposure for corticosterone measurement; samples were taken at the same time points in nonstressed controls.

Blood samples for LH measurement were taken at −2.5 hours and hourly from −2 hours to +2 hours relative to lights out. The next morning, estrous cycle stage was determined, mice were euthanized and the uterus and ovulation were examined as previously described.

### Assays

LH was measured in singlicate in tail blood by the Ligand Assay and Analysis Core of the University of Virginia Center for Research and Reproduction via an ultrasensitive assay (20). The capture monoclonal antibody (antibovine LH beta subunit, 518B7; RRID: AB_2665514) was provided by Janet Roser, University of California. The detection polyclonal antibody (rabbit LH antiserum, AFP240580Rb; RRID: AB_2665533) is provided by the National Hormone and Peptide Program (NHPP). HRP-conjugated polyclonal antibody (goat antirabbit) is purchased from DakoCytomation (Glostrup, Denmark; D048701-2; RRID: AB_2617138). Mouse LH reference prep (AFP5306A; NHPP) is used as the assay standard. The limit of quantitation (functional sensitivity) is defined as the lowest concentration that demonstrates accuracy within 20% of expected values and intra-assay coefficient of variation (%CV) <20% and was determined by serial dilutions of a defined sample pool. Intra-assay %CV is 2.2%. Inter-assay %CVs are 7.3% (low QC, 0.13 ng/mL), 5.0% (medium QC, 0.8 ng/mL) and 6.5% (high QC, 2.3 ng/mL). Functional sensitivity is 0.016 ng/mL. To objectively identify the occurrence of a proestrous LH surge, LH values had to reach ≥3.8 ng/mL, which was the mean + 3SD of LH concentrations measured on the morning of proestrus at 0 hours in prior work (17). Serum corticosterone was determined in duplicate aliquots (2 μL) using the DetectX Corticosterone Enzyme Immunoassay kit (RRID: AB_2877626, Arbor Assays; Ann Arbor, MI, USA). Intra- and inter-assay coefficients of variation for this assay are 5.2% and 7.9%, respectively, and assay sensitivity is 18.6 pg/mL.

### Statistics

Analyses were performed using Prism 11 (GraphPad Software, Boston, MA, USA). Some groups in all analyses were not normally distributed (Shapiro-Wilk). Two-sample tests were thus done with the Mann-Whitney U test and multisample tests with Kruskal-Wallis, followed by Dunn’s post hoc. The remainder of the analyses were with 2- or 3-way ANOVAs, which are sufficiently rigorous for nonnormally distributed data (21); Bonferroni post hoc was used. Tests are indicated in the results or figure legends. All values are denoted as mean ± SEM. Significance was set at *P* < .05; exact *P*-values are reported between *P* = .01 and *P* = .1.

## Results

### Experiment 1: Does ALPS disrupt ovulation?

**Figure 1:**
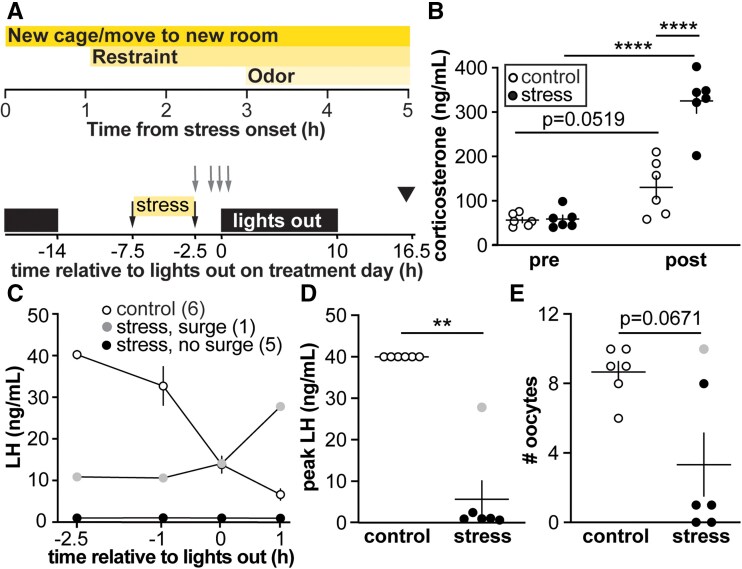
ALPS exposure increases corticosterone and disrupts the proestrous LH surge and ovulation. (A) (top) The layered stress paradigm with time depicted as hours relative to onset of stress. (bottom) Time of blood samples for corticosterone (black arrows) and LH (gray arrows) relative to lights out; mice were euthanized and the presence of oocytes checked the next morning (black arrowhead); dark cycle depicted as black bar. (B) Individual values and mean ± SEM serum corticosterone. (C) Mean ± SEM serum LH over time; groups separated out if criteria for an LH surge were met (value ≥3.8 ng/mL). (D) Individual values and mean ± SEM peak LH value. (E) Individual values and mean ± SEM number of oocytes observed the morning after experiment. Gray circle in panels (C-E) indicates stressed mouse that displayed an LH surge during the sampling window. **P < .01, ****P < .0001; (B) 2-way repeated measures ANOVA/Bonferroni, (D) and (E) Mann-Whitney U test.

Exposure to the ALPS paradigm on proestrus can disrupt the preovulatory LH surge without altering progression of the estrous cycle to estrus (10). While progression of cycle stage is often assumed to indicate ovulation, vaginal cytology is a bioassay for estradiol exposure, not a confirmation of ovulation (22). To test the hypothesis that ALPS blocks ovulation, proestrous mice were exposed to either control or ALPS conditions (n = 6 each, Fig. 1A). There was no difference in pretreatment corticosterone levels between groups (Fig. 1B). Posttreatment corticosterone concentrations in the unstressed controls did not differ from those in the pretreatment sample (*P* = .0519), but the *P*-value did approach that set for significance. Of note, an increase in corticosterone between the pre- and posttreatment samples in unstressed controls would be consistent with the established diurnal change in this hormone and was indeed observed in some later experiments. Posttreatment corticosterone concentrations in ALPS-exposed mice were increased compared to pretreatment values (*P* < .0001). Importantly, posttreatment corticosterone concentrations were higher in the stress group than the controls (*P* < .0001).

All nonstressed control mice had an LH surge within the sampling window (Fig. 1C and 1D), whereas only 1 of 6 ALPS-exposed mice exhibited an LH surge during this time; of note, this was not the mouse with the lowest corticosterone value at the poststress time point. The percentage of nonstressed mice with an LH surge detected was higher than that in stressed animals (*P* = .015, Fisher's exact test). All mice that had an LH surge also had oocytes within the ampulla the following morning (approximately 16-17 hours after the expected LH surge peak), indicating typically timed ovulation (Fig. 1E). The remaining stressed animals did not have an LH surge within the sampling window and exhibited 1 of 2 ovulation responses: no oocytes detected within the oviduct, suggesting blockade of the LH surge and ovulation (n = 2), or oocytes detected outside the ampulla and further along the oviduct (n = 3), suggesting the postulate that ovulation and therefore the LH surge, occurred earlier than expected (23).

### Experiment 2: Does ALPS stimulate LH release resulting in premature ovulation?

**Figure 2:**
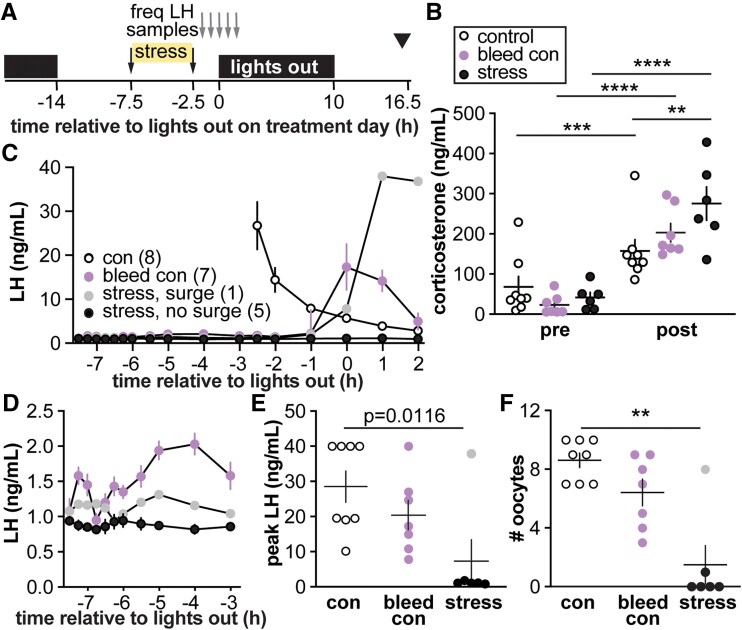
Stress exposure on the morning of proestrus does not increase LH levels. (A) Midmorning on proestrus, mice were either stressed or left under nonstress conditions. Blood samples were taken for corticosterone (black arrows) and LH (gray arrows) throughout the day. Frequent samples for LH measurement were taken during the stress period in stressed mice and at the same time points in bleed control mice; control mice were left undisturbed. Mice were euthanized, and the presence of oocytes was checked the next morning (black arrowhead). (B) Individual values and mean ± SEM serum corticosterone (n = 6-8/each). (C, D) Mean ± SEM serum LH values over time relative to lights out; groups separated out if criteria for an LH surge were met (value ≥3.8 ng/mL); (C) shows the entire experiment, whereas (D) shows the frequent LH sampling period plotted on an expanded *y*-axis. (E) Individual values and mean ± SEM peak LH values. (F) Individual values and mean ± SEM number of oocytes observed the morning after the experiment. Gray-filled circle in panels (C-F) indicates stressed mouse that displayed an LH surge during the sampling window. **P < .01, *** *P* < .001, ****P < .0001; (B) 2-way repeated measures ANOVA/Bonferroni, (E) and (F) Kruskal-Wallis/Dunns.

To test the postulate that application of ALPS stimulated a premature increase in LH sufficient to trigger ovulation, resulting in the premature release of oocytes into the oviduct, we applied the layered stress on the morning of proestrus and measured LH release at various time points (Fig. 2A). There was no difference in pretreatment corticosterone levels between groups (Fig. 2B). All groups exhibited increased corticosterone in the posttreatment sample. There was no difference between the control and bleed control groups during the posttreatment period, and posttreatment corticosterone values were increased in the stress group compared to controls (*P* < .01), but not bleed controls (*P* = .1883).

LH surges were detected in all control and bleed control animals (Fig. 2C and 2D; peak Fig. 2E), and oocytes were within ampullae the following morning (Fig. 2F). In contrast, in ALPS-exposed mice, results were similar to experiment 1, with a variety of responses to stress exposure. An LH surge was detected in 1 of 6 stressed mice, and there were oocytes in the ampulla the following morning. Five ALPS mice had no LH surge; 4 of these had no signs of recently ovulated oocytes within the oviduct, while the remaining mouse had a single oocyte further along in the oviduct the next morning. Stress exposure decreased the percentage of mice having an LH surge within the sampling window when compared to both control groups (*P* < .01, Fisher's exact test). Importantly, during ALPS application, no LH values approaching the surge cutoff were detected in any animal (maximum value observed was 1.3 ng/mL; Fig. 2D), rejecting the hypothesis that an acute stress-induced increase in LH stimulated early ovulation.

### Experiment 3: Can ALPS exposure induce ovulation in the absence of an LH surge?

**Figure 3:**
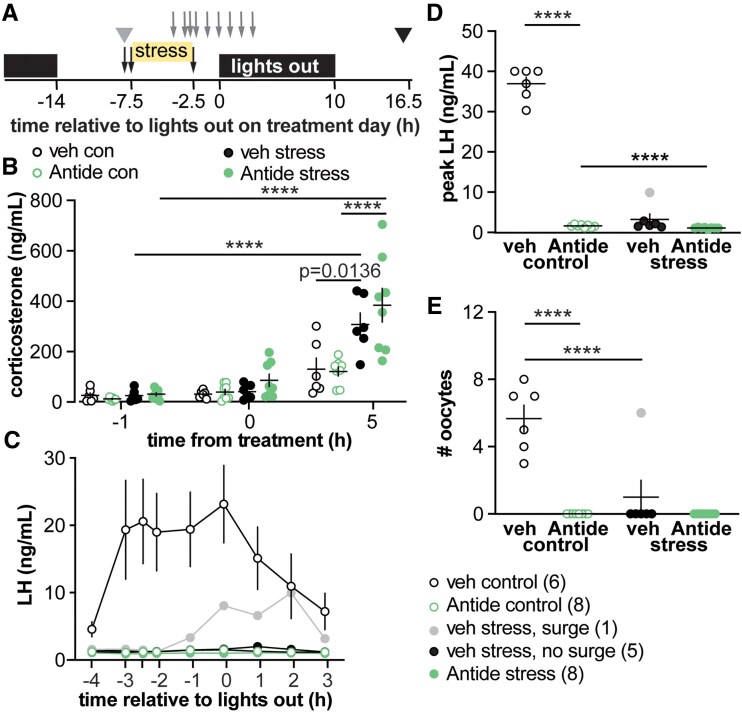
Ovulation in stressed mice does not occur in the presence of a GnRH receptor antagonist. (A) Mice (n = 6-8/group) were assigned to a treatment on the morning of proestrus: vehicle + no stress, Antide + no stress, vehicle + stress, or Antide + stress. Blood samples were taken for corticosterone (black arrows) and LH (gray arrows). Drugs were administered immediately after the first blood sample (gray arrowhead). Ovulation was assessed the following morning (black arrowhead). (B) Individual values and mean ± SEM serum corticosterone. (C) Mean ± SEM LH over time; groups separated out if criteria for an LH surge were met (value ≥3.8 ng/mL). (D) Individual values and mean ± SEM peak LH values. (E) Individual values and mean ± SEM of oocytes observed the following morning. Gray-filled circle in panels (C-E) indicates vehicle + stress mouse that displayed an LH surge during the sampling window. ****P < .0001; (B) 3-way repeated measures ANOVA/Bonferroni, (D) and (E) 2-way ANOVA/Bonferroni.

Results in experiments 1 and 2 suggest that, in some cases, stress exposure appears to trigger premature ovulation without an increase in LH secretion. We tested the hypothesis that exposure to our psychosocial stress paradigm on the morning of proestrus can induce ovulation without increases in circulating LH. To this end, the GnRH receptor antagonist, Antide, was used to block LH release (Fig. 3A).

Basal corticosterone levels (−1 hour time point) were not different between groups (Fig. 3B); corticosterone did not increase after s.c. injection of either vehicle or Antide (preinjection [−1 hour] vs postinjection [0 hours]) (Fig. 3A and 3B). Neither nonstress control group exhibited an increase in circulating corticosterone for the duration of the experiment. Both groups exposed to stress had increased corticosterone at the completion of the stress period compared to their respective prestress value (−1 hour vs 5 hours; both *P*s < .0001). Stress exposure increased corticosterone values at the 5-hour time point in both vehicle- and Antide-treated mice compared to their nonstressed counterparts (vehicle: *P* = .0136; Antide: *P* < .0001).

Vehicle-treated nonstressed mice had a greater percentage of LH surge incidence as compared to the other 3 treatment groups (*P* < .001, Fisher's exact test). All vehicle-treated nonstressed mice (n = 6) had an LH surge near the time of lights out (Fig. 3C and 3D) and showed evidence of ovulation the following morning; all oocytes were contained within a swollen ampulla (Fig. 3E). An LH surge and oocytes were detected in 1 of 6 stressed mice that was given vehicle. The remaining 5 vehicle-treated stressed mice did not have an LH surge within the sampling window or have detectable oocytes in the oviduct the following day, even though progression to estrus occurred as detected by vaginal smear. No mice that received Antide had an LH surge, and no oocytes were detected the following day regardless of stress treatment, rejecting our hypothesis that stressed mice can ovulate without an increase in LH levels.

### Experiment 4: Does adrenalectomy reverse the disruptive effects of ALPS on the LH surge and/or ovulation?

**Figure 4:**
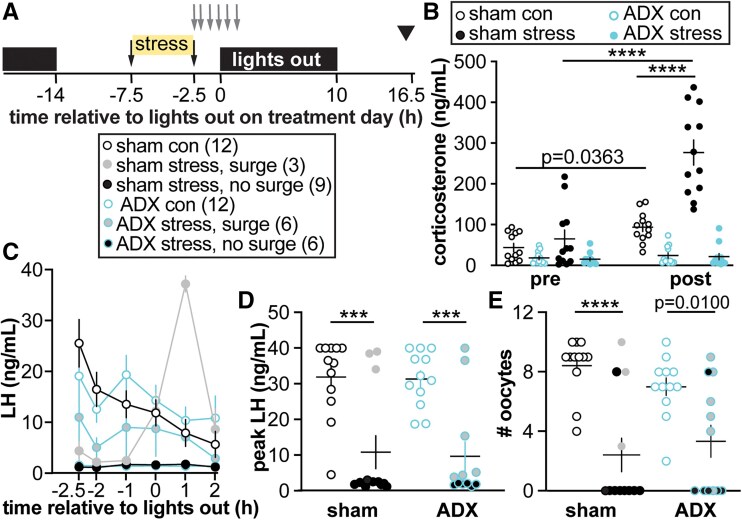
Adrenalectomy does not reverse the stress-induced disruptions of the LH surge and ovulation. (A) Mice (n = 12/group) were assigned to a treatment on the morning of proestrus: sham-operated + no stress, ADX + no stress, sham-operated + stress, or ADX + stress. Blood samples were taken for corticosterone (black arrows) and LH (gray arrows) measurement throughout the experiment. Ovulation was assessed the following morning (black arrowhead). (B) Individual values and mean ± SEM serum corticosterone. (C) Mean ± SEM LH over time; groups separated out if criteria for an LH surge were met (value ≥3.8 ng/mL). (D) Individual values and mean ± SEM peak LH values. (E) Individual values and mean ± SEM of oocytes observed the following morning. Gray-filled circles in panels (C-E) indicate stressed mice that displayed an LH surge during the sampling window. *** *P* < .001, ****P < .0001; (B) 3-way repeated measures ANOVA/Bonferroni, (D) and (E) 2-way ANOVA/Bonferroni.

Stress-related adrenal factors have been implicated in reproductive effects in a variety of species (11, 18, 19). We thus tested the hypothesis that removal of these factors would reverse stress effects on the LH surge and ovulation. Proestrous sham-operated and ADX mice were exposed to control or ALPS conditions (Fig. 4A). There were no differences in pretreatment corticosterone values between groups (Fig. 4B). Posttreatment corticosterone levels were mildly increased in the sham-control group as compared to pretreatment values, but this is consistent with the typical diurnal pattern (*P* = .0363). Posttreatment corticosterone was elevated in sham-operated ALPS mice (pre- vs posttreatment; *P* < .0001) and was higher than the posttreatment levels in the sham-control group (*P* < .0001). No ADX mice, regardless of stress exposure, exhibited a change in corticosterone levels throughout, demonstrating surgical efficacy.

An LH surge on the afternoon of proestrus and oocytes found within the ampullae on the morning of estrus were observed in all nonstressed control animals, regardless of adrenal status (Fig. 4C-4E). There was no difference in peak LH values between sham-operated and ADX controls (Fig. 4D); the number of oocytes harvested from the ampullae from both nonstressed groups was similar (Fig. 4E). In sham-operated stress mice, 8 of 12 (67%) had no detected LH surge or ovulation. Three mice (25%) had peak LH values similar to those observed in the nonstressed groups and ovulated (Fig. 4C-4E). One sham-operated stress mouse (8.3%) had 8 oocytes observed in the ampulla the next day, but no LH surge was detected in the sampling window. In ADX stress mice, the LH surge and oocytes were observed in 5 of 12 animals (41.5%) (Fig. 4C-4E). One mouse had an LH surge, but no oocytes detected; 1 mouse had oocytes detected but had no LH surge (peak LH: 4.4 ng/mL, 0 oocytes; peak LH: 1.87 ng/mL, 8 oocytes, respectively). The remaining 5 animals had neither an LH surge nor oocytes detected (41.7%). The percentage of stressed mice with a normally timed LH surge and ovulation did not differ between sham and ADX mice (*P* = .67, Fisher's exact test), rejecting the hypothesis that removal of the adrenal gland would ameliorate the effects of ALPS treatment.

### Experiment 5: Do stress-like levels of corticosterone mimic the effects of ALPS on the LH surge and/or ovulation?

**Figure 5:**
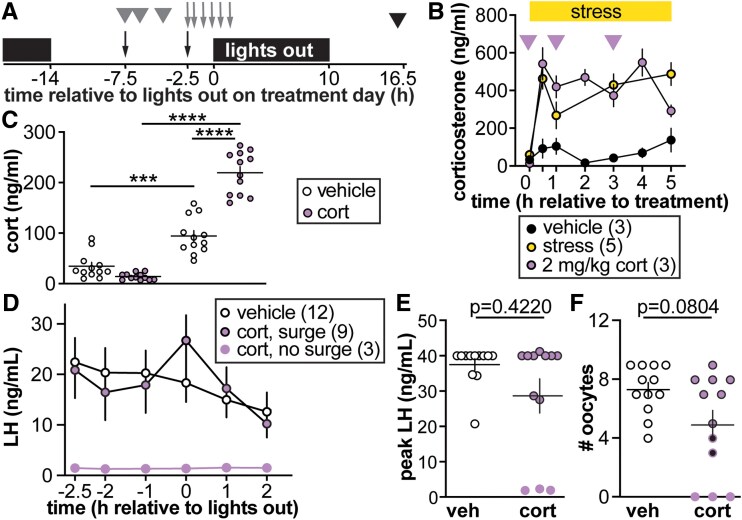
Stress-like levels of exogenous corticosterone do not disrupt the LH surge and ovulation. (A) Mice (n = 12/group) were administered either vehicle or 2 mg/kg corticosterone on the morning of proestrus (administration time points denoted by gray arrowheads). Blood samples to measure corticosterone (black arrows) and LH (gray arrows) were taken throughout the experiment. Ovulation was assessed the following morning (black arrowhead). (B) Pattern of serum corticosterone in mice fed vehicle, 2 mg/kg corticosterone (cort) or exposed to the ALPS paradigm starting at 0 hours through 5 hours (yellow bar). Purple arrowheads indicate time of feeding of vehicle or corticosterone in 60 mg Nutella. (C) Individual values and mean ± SEM serum corticosterone. (D) Mean ± SEM LH over time; groups separated out if criteria for an LH surge were met (value ≥3.8 ng/mL). (E) Individual values and mean ± SEM peak LH values. (F) Individual values and mean ± SEM of oocytes observed the following morning. Black-filled circles are the number of oocytes observed in 1 oviduct only; no correction was made for statistical purposes. ***P < .001, ****P < .0001; (C) 2-way repeated measures ANOVA/Bonferroni, (E) and (F) Mann-Whitney U test.

We next sought to investigate the potential role of a critical adrenal steroid involved in stress response: corticosterone. Mice were assigned to 1 of 2 treatment groups (n = 12/group): vehicle control or 2 mg/kg corticosterone given orally via Nutella. In both groups, Nutella was given at 3 different time points: 0, 1, 3 hours (time relative to first administration) (Fig. 5A). This dose and administration protocol produces a circulating corticosterone profile similar to what is observed during exposure to ALPS (Fig. 5B).

Prior to any treatment, basal corticosterone levels were not different between groups (Fig. 5C). Upon completion of the treatment period, increased corticosterone values were seen in both vehicle- and corticosterone-treated animals as compared to pretreatment values (vehicle: *P* < .001; corticosterone: *P* < .0001). Importantly, the corticosterone-treated group had increased corticosterone levels compared to vehicle-treated mice during the posttreatment period (*P* < .0001). As noted with previous experiments, the increase observed in the vehicle-treated mice is similar to the normal diurnal pattern seen in these mice.

The LH surge and ovulation were detected in a similar proportion of vehicle-treated and corticosterone-treated mice (12 of 12 vehicle and 9 of 12 corticosterone; *P* = .22, Fisher's exact test) (Fig. 5D-5F). All mice that had an LH surge observed on the evening of proestrus also had oocytes within a swollen ampulla the following morning. Two mice in the corticosterone-treated group had 1 oviduct damaged during dissection, so values for these animals reflect a single oviduct (Fig. 5F, black-filled circles).

### Experiment 6: Does blockade of β-adrenergic receptors reverse stress-induced disruption of the LH surge and/or ovulation?

**Figure 6:**
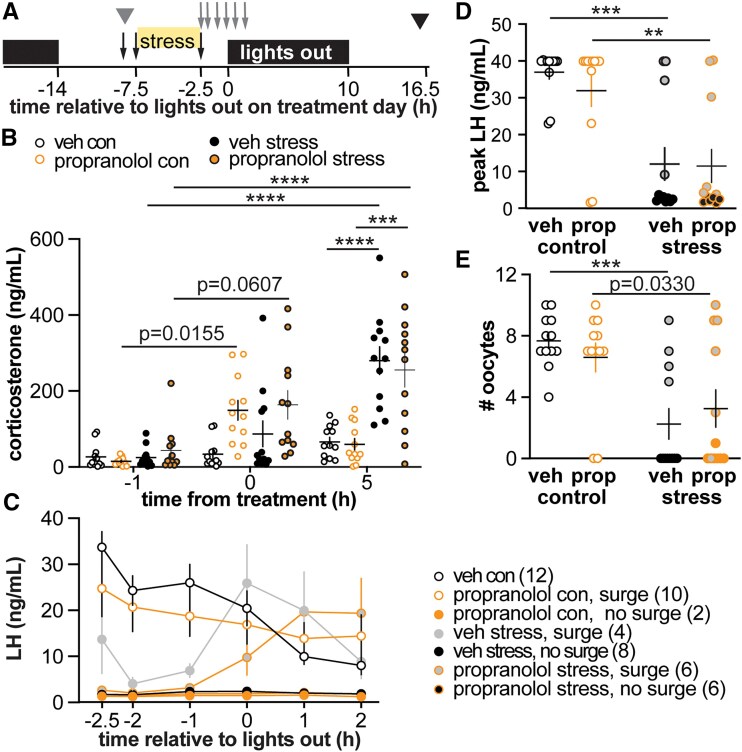
Antagonism of β-adrenergic receptors does not rescue the suppressive effects of psychosocial stress on the LH surge and/or ovulation. (A) Mice (n = 12/group) were assigned to a treatment on the morning of proestrus: vehicle + no stress, propranolol + no stress, vehicle + stress, or propranolol + stress. Drugs were administered immediately after the first blood sample (gray arrowhead). Blood samples to measure corticosterone (black arrows) and LH (gray arrows) were taken throughout the experiment. Ovulation was assessed the following morning (black arrowhead). (B) Individual values and mean ± SEM serum corticosterone. (C) Mean ± SEM LH over time; groups separated out if criteria for an LH surge were met (value ≥3.8 ng/mL). (D) Individual values and mean ± SEM peak LH values. (E) Individual values and mean ± SEM of oocytes observed the following morning. Gray-filled circles in panels (C-E) indicate stressed mice that displayed an LH surge during the sampling window. **P < .01, *** *P* < .001, **** *P* < .0001; (B) 3-way repeated measures ANOVA/Bonferroni, (D) and (E) 2-way ANOVA/Bonferroni.

To further investigate whether adrenal factors play a role in the suppressive effects of psychosocial stress on the preovulatory LH surge and/or ovulation, we next sought to test whether blocking β-adrenergic signaling, which can play a role in the suppressive effects of catecholamines on the HPG axis (24), can reverse these negative reproductive outcomes. On the morning of proestrus, mice were assigned 1 of 4 treatments (n = 12/group): vehicle + no stress, vehicle + stress, 5 mg/kg propranolol (a nonselective β-adrenergic receptor antagonist) + no stress or 5 mg/kg propranolol + stress. Vehicle or propranolol was administered 1 hour prior to onset of stress (Fig. 6A).

No increase in corticosterone was observed in the vehicle-treated mice, suggesting s.c. injection itself did not elicit a stress response (preinjection [−1 hour] vs postinjection [0 hours]) (Fig. 6B). The propranolol-treated mice showed an increase in corticosterone after administration in nonstressed mice and approached the threshold for significance in the stressed group (pre- [−1 hour] vs postinjection [0 hours]: propranolol + no stress, *P* = .0155; propranolol + stress, *P* = .0607) (Fig. 6B). Corticosterone values decreased to levels similar to vehicle control mice at 5 hours in the propranolol + no stress group but remained elevated in mice exposed to the stress paradigm (propranolol: no stress vs stress, *P* < .001), indicating the propranolol-treated stressed mice were responding to ALPS. In the posttreatment period, corticosterone values in mice kept under nonstress conditions were lower than those exposed to stress (nonstress vs stress: vehicle, *P* < .0001; propranolol, *P* < .001).

All vehicle-treated nonstressed mice displayed an LH surge within the sampling window, and oocytes were observed in ampullae the following morning (Fig. 6C-6E). Surprisingly, there were 2 (of 12) propranolol-treated control mice that did not have an LH surge or oocytes detected; LH surges and oocytes were observed in the remaining 10 mice (Fig. 6C-6E). Four of 12 vehicle + stress mice had a late-afternoon surge with oocytes observed in ampullae the next day. Of the remaining 8 vehicle + stress animals, no LH surges or ovulation were detected (Fig. 6C-6E). Similarly, LH surges were observed in 6 of 12 propranolol-treated stressed animals; 5 had oocytes in ampullae the next morning (one mouse did not ovulate with a peak LH of 4.3 ng/mL). Of the 6 (of 12) propranolol + stress mice that did not have an LH surge within the sampling window, 1 showed evidence of ovulation the next morning (1 oocyte in an ampulla) (Fig. 6C-6E). The number of animals that ovulated overnight was similar in stressed mice regardless of treatment: 4 of 12 vehicle + stress vs 6 of 12 propranolol + stress (*P* = .68, Fisher's exact test).

## Discussion

A model of ALPS disrupts the LH surge in a majority of mice when applied on the morning of proestrus (10). These observations raised the intriguing question of whether mice ovulated after ALPS exposure. Here, we showed that stress can affect the LH surge and ovulation in multiple ways: no disruption, mistiming, or complete blockade. Removal of the adrenal gland did not reverse the effects of stress, nor did disruption of β-adrenergic signaling. Similarly, administration of stress-like levels of corticosterone in the absence of stress failed to affect the LH surge and ovulation.

These findings support the disruption of the LH surge by ALPS on the morning of proestrus and extend them to examine ovulation. Surprisingly, in experiments 1 and 2, there were several stress-exposed mice that appeared to ovulate sooner than expected. In these animals, oocytes were not observed in the ampulla the following morning; rather, they had progressed further down the oviduct (23), suggesting the hypothesis that ALPS can induce early ovulation in some cases. There is evidence that acute bouts of stress can increase LH in both male and female rats (25-27); effects on ovulation in the latter are lacking. In women, there is some evidence that acute stress may induce ovulation as pregnancy rates as a result of rape are reported to be consistently higher than rates subsequent to consensual intercourse (28). We thus tested the hypothesis that ALPS increases LH levels. With our sampling paradigm, we did not observe an increase in LH that approached the minimum value for surge definition in any animal during stress exposure, leading us to reject this hypothesis.

The question remains: why do some mice show evidence of early ovulation? It is possible that our sampling paradigm was not frequent enough to catch small increases in LH, but with its 20-minute half-life, that seems unlikely. It is well-accepted that an increase in LH must precede ovulation, but there is at least 1 report that this is not necessarily the case (29). Ovulation-related changes in cyclic nucleotide signaling in granulosa cells occur within minutes of LH treatment of mouse preovulatory follicles in vitro (30). The ovulatory process involves many steps, some of which are initiated before a peak LH surge is observed (31). To this end, we tested if ALPS could induce ovulation in the absence of increases in circulating LH by administering the GnRH-receptor antagonist, Antide (experiment 3). No Antide-treated animal had an LH surge or evidence of ovulation after ALPS application. This is consistent with reports that *Lhcgr* null mice and women with inactivating mutations of LHCGR do not ovulate (32-35). These genotypes have not been paired with a stress stimulus, and it remains possible that ALPS increases another factor that induces ovulation proximal to the time of stress application in a subset of animals. In this regard, FSH can influence when ovulation occurs. FSH levels increase during periods of stress (27, 36, 37), and recombinant FSH induced ovulation in hypophysectomized rats (38). Antide also reduces FSH release, but less completely and with a slower time course (39); while no Antide-treated mouse ovulated after ALPS exposure, we cannot eliminate FSH as a possible factor. Additionally, both acute stress and central administration of corticotropin-releasing hormone have been shown to increase circulating prolactin levels (40, 41), which would typically inhibit ovulation, but some reports observe decreases that could be potentially permissive (26, 37).

One critical aspect of stress response is the adrenal gland, which is a component of both the sympathetic nervous system, the immediate “fight or flight” response, and the hypothalamic-pituitary-adrenal axis, which regulates long-term responses to a stressful stimulus. To determine the potential role of adrenal factors in the deleterious effects of ALPS on the LH surge and ovulation, we tested whether adrenalectomy could reverse these stress-induced outcomes (experiment 4). There were no differences in the incidence of the LH surge and/or ovulation in ADX mice after ALPS treatment. In ADX female rhesus monkeys, corticotropin-releasing hormone (CRH) inhibits gonadotropin secretion, suggesting the adrenal gland is not necessary for stress to have effects on the reproductive system (42). These results do not preclude the possibility of effects of specific adrenal factors, however.

In this regard, there has been little study of effects of short-term glucocorticoid treatment on the reproductive outcomes considered in the present study. Most work has been done to investigate effects of chronic administration. For example, long-term (27 hours) cortisol treatment reduced GnRH/LH pulse frequency in ewes, but only in the presence of estradiol (13). Likewise, chronic administration of corticosterone decreased LH pulse frequency in estradiol-treated ovariectomized mice, but not those without estradiol replacement (12). We thus tested whether acute stress-like levels of corticosterone, the final output of hypothalamic-pituitary-adrenal axis activation, in an environment of elevated estradiol levels (ie, proestrus) could recapitulate the effects of ALPS in the absence of stress (experiment 5). Pilot work was conducted to determine a dose and treatment frequency of corticosterone that mimicked levels seen during ALPS. A 5-hour elevation in corticosterone levels, alone, did not alter the incidence of either the LH surge or ovulation. Of interest in this regard, a recent paper showed that when neurons producing RFRP-3 (gonadotropin-inhibitory hormone [GnIH]) are ablated, the estradiol-induced LH surge in ovariectomized mice is not disrupted despite a rise in corticosterone (43).

Other adrenal factors that play a role in stress response are the catecholamines, NE and epinephrine, which could link the reproductive and stress axes. NE is of particular interest as levels are stimulated by estradiol and increase in the brain during the afternoon of proestrus concurrent with LH surge onset (44). Administration of NE into the paraventricular nucleus of the hypothalamus suppressed LH pulse frequency to a greater extent in ovariectomized rats treated with estradiol vs those without. This effect was mediated by CRH, as a CRH antagonist reversed this suppression, implicating NE in stress-induced disruption of gonadotropin secretion (19). Increased estradiol levels have been reported to shift NE signaling from the inhibitory β-adrenergic pathway to the stimulatory α1-adrenergic pathway (45). ALPS interferes with estradiol positive feedback action, as it blocks the estradiol-induced LH surge (10); therefore, a possible mechanism by which this occurs is that the estradiol-mediated switch from β- to α1-adrenergic signaling is disrupted, resulting in suppression of LH secretion. Here, we sought to test if blockade of β-adrenergic signaling would ameliorate the effects of ALPS exposure on the LH surge and ovulation (experiment 6). Administration of propranolol, a nonselective β-adrenergic receptor antagonist, prior to stress onset did not have an effect on the incidence of the LH surge and ovulation as compared to vehicle-treated animals. Propranolol treatment itself increased corticosterone levels but, as seen with previous studies, this did not correlate with disruption of the LH surge or ovulation in nonstressed mice, only in animals exposed to ALPS. Taken together, increased corticosterone and disruptions to β-adrenergic signaling do not mediate the effects of stress seen on these reproductive outcomes.

This begs the question: what else could be mediating the disruptive effects of ALPS on the LH surge and ovulation? Endogenous opioid peptides are a possibility. Endogenous opioids play a role in both the stress and reproductive axes. For example, naloxone, a nonspecific opioid receptor antagonist, reversed hypoglycemia-induced suppression of LH pulses in rats (46). Naloxone treatment also prevented stress-induced suppression of mean LH levels in estradiol-treated ovariectomized marmosets (47). Another factor that is involved in the regulation of both the reproductive and stress axes is GnIH. Glucocorticoid receptors are expressed in a proportion of GnIH-expressing neurons (48), and GnIH neurons can directly interact with GnRH neurons (49, 50) to inhibit action potential firing (51). It is possible that stress acts to increase GnIH levels via glucocorticoid receptors, which then, in turn, suppress GnRH, leading to further disruptions of LH and ovulation. Lastly, stress-induced CRH neuronal activation may be suppressing the LH surge independent of the resulting increases in corticosterone release from the adrenal gland (52). Direct application of CRH suppressed GnRH neuron firing activity in an estradiol-dependent manner (53), and intracerebroventricular infusion of CRH decreased LH surge amplitude in proestrous rats (54). The potential roles of endogenous opioids, GnIH, and CRH warrant further investigation.

In summary, a model of acute psychosocial stress can impact the LH surge and ovulation in a variety of ways: no effect, mistiming or complete blockade. In a small subset of animals, stress appears to induce ovulation, but this is independent of a marked concurrent increase in LH. Additionally, the adrenal gland does not appear to play a role in these suppressive effects, as the removal of the adrenal gland does not reverse the stress-induced disruptions of the LH surge or ovulation, nor do the adrenal factors corticosterone and catecholamine signaling through β-adrenergic receptors. Future studies are needed to further tease out potential mediators and mechanisms of this stress response on the reproductive axis.

## Data Availability

Original data generated and analyzed during this study are included in this published article.

## Abbreviations

ADX: adrenalectomy
ALPS: acute layered psychosocial stress
CRH: corticotropin-releasing hormone
GnIH: gonadotropin-inhibitory hormone
NE: norepinephrine
